# Birth weight and nutritional status of children under five in sub-Saharan Africa

**DOI:** 10.1371/journal.pone.0269279

**Published:** 2022-06-09

**Authors:** Richard Gyan Aboagye, Bright Opoku Ahinkorah, Abdul-Aziz Seidu, James Boadu Frimpong, Anita Gracious Archer, Collins Adu, John Elvis Hagan, Hubert Amu, Sanni Yaya

**Affiliations:** 1 Department of Family and Community Health, School of Public Health, University of Health and Allied Sciences, Hohoe, Ghana; 2 School of Public Health, Faculty of Health, University of Technology Sydney, Sydney, Australia; 3 Center for Gender and Advocacy, Takoradi Technical University, Takoradi, Ghana; 4 College of Public Health, Medical and Veterinary Sciences, James Cook University, Townsville, Australia; 5 Department of Health, Physical Education, and Recreation, University of Cape Coast, Cape Coast, Ghana; 6 School of Nursing and Midwifery, University of Health and Allied Sciences, Ho, Ghana; 7 Department of Health Promotion, Education and Disability Studies, Kwame Nkrumah University of Science and Technology, Kumasi, Ghana; 8 Neurocognition and Action-Biomechanics-Research Group, Faculty of Psychology and Sport Sciences, Bielefeld University, Bielefeld, Germany; 9 Department of Population and Behavioural Sciences, School of Public Health, University of Health and Allied Sciences, Hohoe, Ghana; 10 School of International Development and Global Studies, University of Ottawa, Ottawa, Canada; 11 The George Institute for Global Health, Imperial College London, London, United Kingdom; Food and Drug Administration, UNITED STATES

## Abstract

**Introduction:**

Over the past three decades, undernutrition has become a major cause of morbidity and mortality among children under five years globally. Low birth weight has been identified as a risk factor for child morbidity and mortality, especially among children under five years in sub-Saharan Africa. There is, however, a paucity of empirical literature establishing the association between low birth weight and undernutrition in sub-Saharan Africa. We examined the association between birth weight and nutritional status of children under five in sub-Saharan Africa.

**Methods:**

Our analyses were performed on a weighted sample of 110,497 children under five years from 32 countries in sub-Saharan Africa. Data were obtained from the Demographic and Health Surveys conducted from 2010 to 2019. We reported the prevalence of low birth weight and nutritional status (stunting, wasting, and underweight) for all the 32 countries using percentages. We used multilevel binary logistic regression to examine the association between birth weight and nutritional status (stunting, wasting, and underweight) of the children, controlling for covariates. The results of the regression analyses were presented using adjusted odds ratios (aOR) with 95% confidence intervals. Statistical significance was set at p<0.05.

**Results:**

The prevalence of low birth weight was 5.4%, with the highest (13.1%) and lowest (0.9%) reportedin South Africa and Chad, respectively. The pooled prevalence of wasting, underweight, and stunting were 8.1%, 17.0%, and 31.3%, respectively. Niger had the highest prevalence of wasting (21.5%) and underweight (37.1%), whereas Burundi had the highest prevalence of stunting (51.7%). We found that children with low birth weight were more likely to be stunted [aOR = 1.68, 95% CI = 1.58–1.78], underweight [aOR = 1.82, 95% CI = 1.70–1.94], and wasted [aOR = 1.35, 95% CI = 1.20–1.38] after controlling for covariates.

**Conclusion:**

Our study has demonstrated that low birth weight is a key determinant of undernutrition among children under five in sub-Saharan Africa. Policymakers need to give special attention to improving the nutritional status of children under-five years in sub-Saharan Africa by implementing measures aimed at enhancing the weight of children. To accelerate progress towards the achievement of the Sustainable Development Goal 3.2 target of ending preventable deaths of newborns and under-five by 2030, it is imperative for countries in sub-Saharan Africa to intensify interventions aimed at improving maternal and child nutrition. Specific nutrition interventions such as dietary modification counselling should prioritized.

## Introduction

Over the past three decades, malnutrition has become a major cause of morbidity and mortality among children under five years [1]. Malnutrition has both short and long-term consequences which pose serious health issues to the smooth growth and development of children [2–5]. Research shows that compared with well-nourished children, malnourished children are physically, intellectually, and emotionally under productive and are at a higher risk of experiencing chronic ailments and disabilities [6–9]. Additionally, malnutrition has been identified as one of the causes of anthropometric deficits in children under the age of five in most low- and middle-income countries [LMICs] [10, 11].

Despite the availability interventions aimed eliminating undernutrition among children, the phenomenon remains a health burden LMCIs [12–15]. In sub-Saharan Africa (SSA), for instance, stunting, wasting, and underweight are highest in Burundi (57.7%), Niger (18.0%), and Burundi (28.8%) [12]. This accentuates the continuous need to direct the focus of research to ascertain the factors that account for the high rates of undernutrition among children under-five years, and to design appropriate interventions to avert the trend.

Low birth weight has also been identified as a risk factor for child morbidity and mortality especially among children under age five [16–19]. A study in Bangladesh revealed that children with low birth weight were at a higher risk of experiencing undernutrition compared to their normal birth weight peers [20]. Previous studies also found child’s age, mother’s educational level, low birth weight, household wealth index, place of residence, low maternal height, type of toilet facility, and type of cooking oil as associated factors of undernutrition among children under five years [12, 20–24].

Notwithstanding, an exclusive search of the available literature showed that up until now, no study has examined the association between low birth weight and undernutrition indicators among children under-five years using nationally representative data in SSA. This makes it difficult for policymakers and health organizations to develop and implement policies, programs, and interventions aimed at reducing the rates and the detrimental effects of undernutrition among children under age five at the sub-regional level. This presents a wide research gap, which this study seeks to address.

Therefore, the study assessed the association between birth weight and nutritional status (stunting, wasting, and underweight) of children under-five years using data from Demographic and Health Surveys (DHS) conducted in 32 countries in SSA. The findings of this study are expected to assist direct policies, programmes, and interventions that will lead to a reduction in the prevalence of undernutrition in SSA.

## Materials and methods

### Data source and study design

This study involved cross-sectional analyses of data from the DHS of 32 countries in SSA. Only the data from the most recent surveys (2010–2019) from these countries were analysed. Specifically, we utilised data from the Child’s Recode (KR File) from the 32 countries. DHS is a countrywide representative survey conducted to collect data on health indicators such as nutrition, maternal, and child health among others [25]. Since the inception of the DHS, it has practically been conducted in over 85 LMICs globally [25]. The DHS utilised a two-stage sampling technique to recruit respondents for the study. A detailed explanation of the data collection methods and sampling techniques has been published elsewhere [26]. A total of 110,497 children under five years with complete cases of variables of interest were included in the study. Table 1 shows a description of the study sample included in the analysis per country.

**Table 1:** Description of study sample.

Countries	Year of survey	Weighted N	Weighted %
1. Angola	2015–16	3,538	3.20
2. Burkina Faso	2010	4,474	4.05
3. Benin	2017–18	7,686	6.96
4. Burundi	2016–17	4,178	3.78
5. DR Congo	2013–14	4,739	4.29
6. Congo	2011–12	2,589	2.34
7. Cote d’Ivoire	2011–12	2,106	1.91
8. Cameroon	2018	2,863	2.59
9. Ethiopia	2016	6,553	5.93
10. Gabon	2012	1,932	1.75
11. Ghana	2014	1,901	1.72
12. Gambia	2013	752	0.68
13. Guinea	2018	2,212	2.00
14. Kenya	2014	6,016	5.44
15. Comoros	2012	1,276	1.16
16. Liberia	2013	1,959	1.77
17. Lesotho	2014	1,011	0.92
18. Mali	2018	5,437	4.92
19. Malawi	2015–16	4,007	3.63
20. Nigeria	2018	7,322	6.63
21. Niger	2012	3,033	2.74
22. Namibia	2013	892	0.81
23. Rwanda	2014–15	1,346	1.22
24. Sierra Leone	2019	2,657	2.40
25. Senegal	2010–11	1,943	1.76
26. Chad	2014–15	5,879	5.32
27. Togo	2013–14	2,209	2.00
28. Tanzania	2015–16	6,038	5.46
29. Uganda	2016	2,852	2.58
30. South Africa	2016	854	0.77
31. Zambia	2018	6,249	5.66
32. Zimbabwe	2015	3,994	3.61
**All countries**		**110,497**	**100.0**

### Study variables

#### Outcome variables

Three anthropometric indices (stunting [height-for-age z-scores], wasting [weight-for-height z-scores], and underweight [weight-for-age z-scores]) were the outcome variables in ourstudy. The classification of child’s nutritional status was informed by the World Health Organization’s Growth Reference for children under-five years [27].

In classifying stunting, children with height-for-age z-scores below minus 2 (−2.0) standard deviations (SD) less than the mean on the reference standard (moderately or severely stunted) and children with height-for-age z-scores below minus 3 (−3.0 SD) less than the mean on the reference standard (severely stunted) were categorised as stunted and was recoded as “1”. Children whose height-for-age z-scores were greater than minus 2 (−2.0 SD) above the mean on the reference standard were categorized as “Normal” and recoded as “0”.

With wasting, children with weight-for-height z-scores below minus 2 (−2.0 SD) (moderately or severely wasting) and those with weight-for-height z-scores below minus 3 (−3.0) standard deviations (SD) (severely wasting) were classified as wasted. Also, those whose weight-for-height z-scores were higher than minus 2 (−2.0 SD) greater than the mean on the reference standard were considered as “Normal”. In the final analysis, those who were classified as normal were recoded as “0” whilst those wasted were recoded as “1”.

Based on the WHO Growth Reference, children with weight-for-age z-scores below minus 2 (−2.0 SD) less than the mean on the reference standard (moderately or severely underweight) and those with weight-for-age z-scores below minus 3 (−3.0 SD) less than the mean (severely underweight) categorized as “underweight” and coded as “1”. Children whose weight-for-age z-scores were higher than minus 2 (−2.0 SD), thus, greater than the mean on the reference standard were regarded as “Normal” and were recoded as “0”.

#### Key explanatory variable

Birth weight was the main explanatory variable. For this study, only numerical birth weight data contained in the DHS survey for the most recent children were considered. The birth weight data were classified into two groups: normal weight (birth weight ≥2500 g) and low birth weight (birth weight <2500 g). Previous studies have used similar classifications [20, 28, 29].

#### Covariates

We included a total of 18 covariates in our study. We selected the variables based on their availability in the DHS dataset and their association with childhood undernutrition from previous studies [20, 28, 30]. The variables were categorised into individual level factors, household factors, and contextual factors. The individual level factors consisted of sex of child (male and female), age of the child (0, 1, 2, 3, and 4), birth order (1, 2–4, and 5+), and perceived size at birth (large, average, and small). Other individual level factors were educational level (no formal education, primary, secondary, and higher), current working status (yes and no), and postnatal checks within 2 months (yes and no) were maintained. maternal age (15–19 and 20–49), antenatal visits during pregnancy (0, 1–3 and 4 or more), place of delivery (home, health facility, other), and marital status (single and married). The household factors consisted of the source of drinking water (improved and unimproved), type of toilet facility (improved and unimproved), household size (small, medium, and large), exposure to media (yes, no), type of cooking fuel (clean and unclean) and wealth index (poorest, poorer, middle, richer, and richest). The contextual factors include the place of residence (urban and rural) and geographical sub-regions (West, East, Central, and Southern).

### Data analyses

First, frequencies and percentages to show the prevalence of low birth weight and undernutrition (stunting, wasting, and underweight) among children under-five years in the selected sub-Saharan African countries were determined. After, we performed cross-tabulation to determine the distribution of each undernutrition indicator across birth weight and the covariates. Also, we used the Pearson’s chi-square test of independence [*χ*^2^] at a p-value of less than 0.05 to show significant variables associated with each undernutrition indicator. Further, a multilevel binary logistic regression analysis to examine the association between birth weight and each undernutrition indicators, controlling for the covariates. Model O showed the variance in each undernutrition indicator attributed to the clustering of the primary sampling units (PSUs) without the explanatory variables. Model I and Model II contained the individual and household level factors, respectively, while Model III contained the contextual level factors. The final model (Model IV) had all the individual, household, and contextual level factors. The Stata command "melogit" was used in fitting these models. We used Akaike’s Information Criterion (AIC) tests for Model comparison. All the results were presented using adjusted odds ratios (aOR) with 95% Confidence Interval (CI). Sample weight (v005/1,000,000) and the ‘svy’ command were used to correct for over and under-sampling, including the complex survey design to improve our findings’ generalizability. The paper followed the Strengthening the Reporting of Observational Studies in Epidemiology (STROBE) reporting guidelines.

#### Ethical consideration

No ethical clearance was sought in this study because the DHS dataset is freely available in the public domain. Prior to the commencement of this study, permission to use the dataset was sought from the Monitoring and Evaluation to Assess and Use Results Demographic and Health Surveys (MEASUREDHS). All ethical consideration concerning the use of secondary dataset for publication were adhered to. Detailed information about the DHS data usage and ethical standards are available at http://goo.gl/ny8T6X.

## Results

### Prevalence of low birth weight, stunting, wasting, and underweight in sub-Saharan Africa

The prevalence of low birth weight in the 32 countries considered in this study was 5.4%, with the highest (13.1%) and lowest (0.9%) in South Africa and Chad, respectively. The prevalence of wasting, underweight, and stunting were 8.1%, 17.0%, and 31.3%, respectively. The highest prevalence of wasting (21.5%) and underweight (37.1%) were found in Niger while the highest prevalence of stunting (51.7%) was found in Burundi (Table 2).

**Table 2:** Prevalence of low birth weight, stunting, wasting, and underweight in sub-Sharan Africa.

Countries	Stunting	Wasting	Underweight	Low birth weight
Angola	34.6	6.0	19.5	5.9
Burkina Faso	31.7	19.1	27.3	7.9
Benin	28.8	5.6	15.7	7.0
Burundi	51.7	6.0	27.7	7.5
DR Congo	36.6	9.9	20.1	5.3
Congo	20.9	6.5	10.5	7.9
Cote d’Ivoire	27.7	9.6	14.4	7.5
Cameroon	27.9	4.2	10.6	4.1
Ethiopia	36.6	11.5	21.8	2.1
Gabon	15.0	3.7	6.0	10.8
Ghana	15.7	5.4	10.4	6.1
Gambia	27.2	15.3	21.6	3.1
Guinea	31.5	9.8	16.5	5.0
Kenya	24.6	3.8	9.6	5.1
Comoros	27.8	11.5	14.2	11.6
Liberia	28.2	7.3	14.8	1.9
Lesotho	29.7	3.4	9.3	6.8
Mali	25.1	10.5	19.1	5.4
Malawi	34.8	3.0	10.7	9.4
Nigeria	35.1	8.4	21.9	2.0
Niger	40.4	21.5	37.1	3.2
Namibia	21.7	9.1	13.2	9.4
Rwanda	36.4	2.7	9.5	5.0
Sierra Leone	28.2	5.9	13.1	3.2
Senegal	26.0	10.5	16.8	8.6
Chad	37.0	15.4	28.9	0.9
Togo	24.4	8.1	15.6	5.2
Tanzania	32.5	5.6	13.2	3.9
Uganda	27.2	4.5	9.6	5.0
South Africa	25.3	1.6	4.8	13.1
Zambia	33.8	4.7	11.3	6.4
Zimbabwe	26.0	4.1	8.1	7.1
**All countries**	**31.3**	**8.1**	**17.0**	**5.4**

### Distribution of stunting, wasting, and underweight across the explanatory variables

Table 3 shows the distribution of stunting, wasting, and underweight across the low birth weight. The results showed significant disparities in stunting, wasting, and underweight across the birth weight. Specifically, stunting was higher among children with below 2.5kg birth weight (42.2%), compared to those with 2.5kg and above (30.7%). Similarly, underweight was higher among children with below 2.5kg birth weight (26.5%), compared to those whose weight was 2.5kg and above (16.5%). Finally, wasting was higher among children whose weight was below 2.5kg (10.5%), compared to those whose weight was 2.5kg and above (8.0%). In terms of the covariates, significant differences were observed in the prevalence of stunting for all the categories of the covariates except mother’s age, current working status, and marital status. All the categories of the covariates showed significant differences for underweight, except mother’s age. For wasting, significant differences were identified within all the categories of the covariates.

**Table 3:** Bivariate analysis birth weight and undernutrition among children in sub-Saharan Africa.

Variables	Weighted N	Weighted %	Stunted	P-value	Underweight	P-value	Wasted	P-value
**Birth Weight**				<0.001		<0.001		<0.001
2500g and above	104,531	94.6	30.7		16.5		8.0	
Below 2.5	5,966	5.4	42.2		26.5		10.4	
**Individual-level factors**								
**Sex of child**				<0.001		<0.001		<0.001
Male	55,780	50.5	34.0		18.5		9.0	
Female	54,717	49.5	28.5		15.5		7.3	
**Age of child**				<0.001		<0.001		<0.001
0	33,702	30.5	16.8		13.0		10.7	
1	31,381	28.4	36.3		19.7		9.5	
2	22,366	20.2	43.4		20.3		6.1	
3	13,917	12.6	37.4		16.7		4.4	
4	9,131	8.3	28.4		15.4		4.4	
**Birth order**				<0.001		<0.001		<0.001
1	21,832	19.8	29.5		14.7		7.3	
2–4	53,012	48.0	29.8		15.8		7.7	
5+	35,653	32.2	34.7		20.3		9.2	
**Size of child at birth**				<0.001		<0.001		<0.001
Large	38,515	34.9	27.0		12.9		6.4	
Average	54,469	49.3	31.4		16.5		8.0	
Smaller	17,513	15.8	40.3		27.8		12.4	
**Mother’s age (years)**				0.642		0.993		0.001
15–19	7,903	7.2	31.0		17.0		9.4	
20–49	102,594	92.8	31.3		17.0		8.0	
**Maternal educational level**				<0.001		<0.001		<0.001
No education	43,126	39.0	37.3		24.4		11.6	
Primary	36,081	32.6	32.8		14.8		6.3	
Secondary	27,574	25.0	22.7		10.0		5.6	
Higher	3,716	3.4	11.0		6.0		4.6	
**Current working status**				0.089		<0.001		<0.001
No	41,318	37.4	30.9		18.0		9.6	
Yes	69,179	62.6	31.5		16.4		7.3	
**Number of antenatal care visits**				<0.001		<0.001		<0.001
0	11,668	10.6	41.8		28.7		13.1	
1–3	37,922	34.3	34.4		18.7		9.0	
4 or more	60,907	55.1	27.3		13.7		6.6	
**Place of delivery**				<0.001		<0.001		<0.001
Home	35,054	31.7	38.4		24.6		11.3	
Health facility	74,154	67.1	27.8		13.4		6.7	
Other	1,289	1.2	34.8		18.9		7.0	
**Postnatal care visits**				<0.001		<0.001		<0.001
No	64,025	57.9	33.3		18.7		8.5	
Yes	46,472	42.1	28.5		14.7		7.6	
**Marital status**				0.974		<0.001		<0.001
Single	14,610	13.2	31.3		14.9		6.3	
Married	95,887	86.8	31.3		17.4		8.4	
**Household factors**								
**Drinking water source**				<0.001		<0.001		0.048
Improved	71,245	64.5	30.4		16.3		8.0	
Unimproved	39,252	35.5	32.9		18.3		8.4	
**Toilet facility**				<0.001		<0.001		<0.001
Improved	48,169	43.6	27.0		12.8		6.3	
Unimproved	62,328	56.4	34.6		20.3		9.6	
**Household size**				<0.001		<0.001		<0.001
Small	48,221	43.6	30.5		15.5		7.4	
Medium	48,987	44.4	31.6		17.6		8.4	
Large	13,289	12.0	32.9		20.5		10.1	
**Mass media exposure**				<0.001		<0.001		<0.001
No	39,034	35.3	38.1		22.3		10.0	
Yes	71,463	64.7	27.5		14.2		7.1	
**Cooking fuel**				<0.001		<0.001		<0.001
Unclean	99,735	90.3	32.8		18.0		8.5	
Clean	10,762	9.7	17.3		8.0		4.9	
**Wealth index**				<0.001		<0.001		<0.001
Poorest	23,557	21.3	38.5		22.3		9.8	
Poorer	23,332	21.1	35.9		19.5		8.5	
Middle	22,302	20.2	32.6		17.5		8.2	
Richer	21,997	19.9	28.5		14.4		7.3	
Richest	19,309	17.5	18.6		10.1		6.4	
**Contextual factors**								
**Place of residence**				<0.001		<0.001		<0.001
Urban	35,910	32.5	23.1		11.6		6.4	
Rural	74,587	67.5	35.2		19.7		9.0	
**Subregions**				<0.001		<0.001		<0.001
West	43,690	39.5	29.6		19.5		10.2	
East	42,509	38.5	33.3		14.1		5.8	
Central	21,541	19.5	31.4		18.7		9.0	
Southern	2,757	2.5	25.8		9.2		4.6	

*p-values obtained from chi-square test

### Association between birth weight and nutritional status of children under five in sub-Saharan Africa

Model IV of Tables 4–6 show the results of the association between birth weight and nutritional Status of children under five. We found that children with low birth weight were more likely to be stunted [aOR = 1.68, 95% CI = 1.58–1.78], underweight [aOR = 1.82, 95% CI = 1.70–1.94], and wasted [aOR = 1.35, 95% CI = 1.20–1.38]. With the covariates, the likelihood of stunting was higher among older children, children with small size at birth, children whose mothers lived in large households, those who live in rural areas, and Central Africa. The likelihood of underweight was higher among older children, children with small size at birth, those who lived in households with unimproved toilet facility, children whose mothers lived in large households, those who live in rural areas, and Central Africa. The odds of wasting were higher among children whose size at birth was small, those who lived in households with unimproved toilet facilities, those whose mothers were of the richest wealth quintile, and those who lived in rural areas.

**Table 4:** Fixed and random effects results on the association between birth weight and stunting among children in sub-Sharan Africa.

	Model O	Model I	Model II	Model III	Model IV
		aOR [95% CI]	aOR [95% CI]	aOR [95% CI]	aOR [95% CI]
**Fixed effects**					
**Birth Weight**					
2500g and above		1.00 [1.00, 1.00]			1.00 [1.00, 1.00]
Below 2.5		1.65*** [1.55, 1.75]			1.68***[1.58, 1.78]
**Sex of child**					
Male		1.00 [1.00, 1.00]			1.00 [1.00, 1.00]
Female		0.75*** [0.71, 0.75]			0.72***[0.70, 0.74]
**Age of child**					
0		1.00 [1.00, 1.00]			1.00 [1.00, 1.00]
1		2.98*** [2.87, 3.09]			3.01***[2.89, 3.12]
2		4.12*** [3.96, 4.29]			4.23***[4.06, 4.40]
3		3.32*** [3.17, 3.48]			3.43***[3.28, 3.60]
4		2.20*** [2.08, 2.33]			2.31***[2.18, 2.44]
**Birth order**					
1		1.00 [1.00, 1.00]			1.00 [1.00, 1.00]
2–4		0.89*** [0.86, 0.93]			0.90***[0.87, 0.94]
5+		0.95** [0.91, 0.98]			0.92***[0.88, 0.96]
**Size of child at birth**					
Large		1.00 [1.00, 1.00]			1.00 [1.00, 1.00]
Average		1.22*** [1.19, 1.26]			1.23***[1.19, 1.27]
Small		1.67*** [1.61, 1.74]			1.67***[1.60, 1.74]
**Maternal educational level**					
No education		1.00 [1.00, 1.00]			1.00 [1.00, 1.00]
Primary		0.90*** [0.88, 0.93]			0.86***[0.83, 0.89]
Secondary		0.59*** [0.56, 0.61]			0.65***[0.63, 0.68]
Higher		0.26*** [0.24, 0.29]			0.40***[0.35, 0.44]
**Number of antenatal care visits**					
0		1.00 [1.00, 1.00]			1.00 [1.00, 1.00]
1–3		0.89*** [0.85, 0.93]			0.92***[0.88, 0.97]
4 or more		0.74*** [0.70, 0.77]			0.79***[0.76, 0.83]
**Place of delivery**					
Home		1.00 [1.00, 1.00]			1.00 [1.00, 1.00]
Health facility		0.80*** [0.77, 0.82]			0.88***[0.85, 0.91]
Others		1.06 [0.94, 1.20]			1.07 [0.94, 1.21]
**Postnatal care visits**					
No		1.00 [1.00, 1.00]			1.00 [1.00,1.00]
Yes		0.87*** [0.85, 0.89]			0.90*** [0.88,0.93]
**Mass media exposure**					
No			1.00 [1.00,1.00]		1.00 [1.00, 1.00]
Yes			0.77***[0.75,0.79]		0.87***[0.84, 0.89]
**Toilet facility**					
Improved			1.00 [1.00,1.00]		1.00 [1.00, 1.00]
Unimproved			1.06***[1.03,1.09]		1.00 [0.97, 1.03]
**Drinking water source**					
Improved			1.00 [1.00,1.00]		1.00 [1.00, 1.00]
Unimproved			0.99 [0.97,1.02]		0.97[0.94, 1.00]
**Household size**					
Small			1.00 [1.00,1.00]		1.00 [1.00, 1.00]
Medium			1.01 [0.98,1.04]		1.01 [0.97, 1.04]
Large			1.10***[1.05,1.14]		1.13***[1.08, 1.19]
**Cooking fuel**					
Unclean			1.00 [1.00,1.00]		1.00 [1.00, 1.00]
Clean			0.64***[0.61,0.68]		0.74***[0.70, 0.79]
**Wealth index**					
Poorest			1.00 [1.00,1.00]		1.00 [1.00, 1.00]
Poorer			0.96 [0.93,1.00]		0.99 [0.95, 1.03]
Middle			0.88***[0.85,0.92]		0.92***[0.88, 0.96]
Richer			0.77***[0.74,0.80]		0.83***[0.79, 0.87]
Richest			0.52***[0.50,0.55]		0.63***[0.59, 0.67]
**Place of residence**					
Urban				1.00 [1.00,1.00]	1.00 [1.00, 1.00]
Rural				1.71***[1.66,1.76]	1.10***[1.06, 1.14]
**Subregions**					
West				1.00 [1.00,1.00]	1.00 [1.00, 1.00]
East				1.10***[1.07,1.13]	1.20*** [1.16, 1.25]
Central				1.19***[1.15,1.24]	1.28*** [1.23, 1.33]
Southern				0.86***[0.79,0.94]	1.15** [1.04, 1.26]
**Random effect result**					
PSU variance (95% CI)	0.017 [0.012, 0.024]	0.012 [0.008, 0.019]	0.013 [0.009, 0.019]	0.014 [0.010, 0.020]	0.011 [0.007, 0.017]
ICC	0.005	0.004	0.004	0.004	0.003
LR Test	69.41 (<0.001)	36.84 (<0.001)	44.93 (<0.001)	49.59 (<0.001)	29.71 (<0.001)
Wald chi-square	Reference	9067.65***	2489.23***	1402.07***	9910.39***
Model fitness					
Log-likelihood	-68760.219	-63436.358	-67399.613	-68030.929	-62842.461
AIC	137524.4	126912.7	134823.2	136073.9	125752.9
N	110497	110497	110497	110497	110497
Number of clusters	1608	1608	1608	1608	1608

aOR adjusted odds ratios; CI Confidence Interval;

* *p* < 0.05,

** *p* < 0.01,

*** *p* < 0.001

1 = Reference category PSU = Primary Sampling Unit; ICC = Intra-Class Correlation; LR Test = Likelihood ratio Test; AIC = Akaike’s Information Criterion

**Table 5:** Fixed and random effects results on the association between birth weight and underweight among children in sub-Sharan Africa.

Variables	Model O	Model I	Model II	Model III	Model IV
		aOR [95% CI]	aOR [95% CI]	aOR [95% CI]	aOR [95% CI]
**Fixed effects**					
**Birth eight**					
2500g and above		1.00 [1.00, 1.00]			1.00 [1.00, 1.00]
Below 2.5		1.79*** [1.67, 1.91]			1.82***[1.70, 1.94]
**Sex of child**					
Male		1.00 [1.00, 1.00]			1.00 [1.00, 1.00]
Sex		0.76*** [0.73, 0.78]			0.75***[0.73, 0.78]
**Age of child**					
0		1.00 [1.00, 1.00]			1.00 [1.00, 1.00]
1		1.74*** [1.67, 1.82]			1.77***[1.69, 1.85]
2		1.84*** [1.76, 1.93]			1.90***[1.81, 1.99]
3		1.51*** [1.42, 1.59]			1.58***[1.49, 1.67]
4		1.36*** [1.27, 1.45]			1.46***[1.36, 1.56]
**Birth order**					
1		1.00 [1.00, 1.00]			1.00 [1.00, 1.00]
2–4		0.96 [0.91, 1.00]			0.97 [0.93, 1.02]
5+		1.03 [0.98, 1.09]			1.02 [0.97, 1.08]
**Size of child at birth**					
Large		1.00 [1.00, 1.00]			1.00 [1.00, 1.00]
Average		1.32*** [1.27, 1.37]			1.37***[1.32, 1.43]
Small		2.19*** [2.09, 2.29]			2.26***[2.16, 2.37]
**Maternal level of education**					
No education		1.00 [1.00, 1.00]			1.00 [1.00, 1.00]
Primary		0.63*** [0.60, 0.65]			0.71***[0.69, 0.75]
Secondary		0.45*** [0.43, 0.48]			0.60***[0.53, 0.59]
Higher		0.28*** [0.24, 0.32]			0.44***[0.38, 0.51]
**current working status**					
No		1.00 [1.00, 1.00]			1.00 [1.00, 1.00]
Yes		0.95** [0.92, 0.98]			0.92***[0.89, 0.95]
**Marital status**					
Single		1.00 [1.00, 1.00]			1.00 [1.00, 1.00]
Married		0.98 [0.93, 1.03]			0.97 [0.92, 1.02]
**Number of antenatal care visits**					
0		1.00 [1.00, 1.00]			1.00 [1.00, 1.00]
1–3		0.79*** [0.75, 0.83]			0.84***[0.79, 0.88]
4 or more		0.68*** [0.65, 0.72]			0.74***[0.70, 0.78]
**Place of delivery**					
Home		1.00 [1.00, 1.00]			1.00 [1.00, 1.00]
Health facility		0.67*** [0.65, 0.70]			0.74***[0.71, 0.77]
Others		0.94 [0.82, 1.09]			1.03 [0.89, 1.19]
**Postnatal care visits**					
No		1.00 [1.00, 1.00]			1.00 [1.00, 1.00]
Yes		0.90*** [0.87, 0.93]			0.93***[0.90, 0.96]
**Mass media exposure**					
No			1.00 [1.00, 1.00]		1.00 [1.00, 1.00]
Yes			0.69***[0.67, 0.72]		0.84***[0.81, 0.87]
**Toilet facility**					
Improved			1.00 [1.00, 1.00]		1.00 [1.00, 1.00]
Unimproved			1.33***[1.28, 1.38]		1.11***[1.06, 1.15]
**Drinking water source**					
Improved			1.00 [1.00, 1.00]		1.00 [1.00, 1.00]
Unimproved			1.00 [0.97, 1.04]		0.95**[0.91, 0.98]
**Household size**					
Small			1.00 [1.00, 1.00]		1.00 [1.00, 1.00]
Medium			1.10***[1.07, 1.14]		1.01 [0.98, 1.05]
Large			1.33***[1.27, 1.40]		1.10***[1.05, 1.16]
**Cooking fuel**					
Unclean			1.00 [1.00, 1.00]		1.00 [1.00, 1.00]
Clean			0.57***[0.53,0.62]		0.69***[0.64,0.76]
**Wealth index**					
Poorest			1.00 [1.00, 1.00]		1.00 [1.00, 1.00]
Poorer			0.92***[0.88, 0.96]		0.96 [0.92, 1.00]
Middle			0.90***[0.86, 0.95]		0.96 [0.91, 1.01]
Richer			0.84***[0.79, 0.88]		0.94*[0.89, 0.99]
Richest			0.69***[0.64, 0.73]		0.91**[0.84, 0.97]
**Place of residence**					
Urban				1.00 [1.00, 1.00]	1.00 [1.00, 1.00]
Rural				1.85***[1.78, 1.92]	1.17***[1.12, 1.23]
**Subregions**					
West				1.00 [1.00, 1.00]	1.00 [1.00, 1.00]
East				0.66***[0.63, 0.68]	0.76***[0.73, 0.79]
Central				1.04 [1.00, 1.08]	1.10***[1.05, 1.15]
Southern				0.42***[0.37, 0.48]	0.66***[0.57, 0.76]
**Random effect result**					
PSU variance (95% CI)	0.021 [0.015, 0.031]	0.012 [0.007, 0.021]	0.017 [0.011, 0.026]	0.024 [0.017, 0.034]	0.015 [0.009, 0.024]
ICC	0.006	0.004	0.005	0.007	0.005
LR Test	52.35 (<0.001)	18.83 (<0.001)	35.22 (<0.001)	60.01 (<0.001)	26.58 (<0.001)
Wald chi-square	Reference	6415.28***	2167.25***	1649.01***	6887.51***
Model fitness					
Log-likelihood	-51178.564	-47677.097	-49984.975	-50289.332	-47322.165
AIC	102361.1	95398.19	99993.95	100590.7	94716.33
N	110497	110497	110497	110497	110497
Number of clusters	1608	1608	1608	1608	1608

aOR adjusted odds ratios; CI Confidence Interval;

* *p* < 0.05,

** *p* < 0.01,

*** *p* < 0.001;

1 = Reference category PSU = Primary Sampling Unit; ICC = Intra-Class Correlation; LR Test = Likelihood ratio Test; AIC = Akaike’s Information Criterion

**Table 6:** Fixed and random effects results on the association between birth weight and wasting among children in sub-Saharan Africa.

Variables	Model O	Model I	Model II	Model III	Model IV
		aOR [95% CI]	aOR [95% CI]	aOR [95% CI]	aOR [95% CI]
**Fixed effects**					
**Birth Weight**					
2500g and above		1.00 [1.00, 1.00]			1.00 [1.00, 1.00]
Below 2.5		1.35*** [1.23, 1.48]			1.35***[1.23, 1.48]
**Sex of child**					
Male		1.00 [1.00, 1.00]			1.00 [1.00, 1.00]
Female		0.74*** [0.71, 0.78]			0.741***[0.71, 0.77]
**Age of child**					
0		1.00 [1.00, 1.00]			1.00 [1.00, 1.00]
1		0.91** [0.87, 0.96]			0.92**[0.87, 0.97]
2		0.55*** [0.51, 0.58]			0.55***[0.52, 0.59]
3		0.41*** [0.37, 0.44]			0.41***[0.38, 0.45]
4		0.43*** [0.38, 0.47]			0.44***[0.40, 0.49]
**Birth order**					
0		1.00 [1.00,1.00]			1.00 [1.00, 1.00]
2–4		1.02 [0.95, 1.09]			1.02 [0.95, 1.10]
5+		1.07 [0.99, 1.16]			1.07 [0.98, 1.15]
**Size of child at birth**					
Large		1.00 [1.00, 1.00]			1.00 [1.00,1.00]
Average		1.24*** [1.18, 1.31]			1.29***[1.23, 1.36]
Small		1.79*** [1.68, 1.91]			1.85***[1.73, 1.97]
**Maternal age (years)**					
15–19		1.00 [1.00, 1.00]			1.00 [1.00, 1.00]
20–49		0.96 [0.87, 1.05]			0.99 [0.91, 1.09]
**Maternal educational level**					
No education		1.00 [1.00, 1.00]			1.00 [1.00, 1.00]
Primary		0.58*** [0.55, 0.61]			0.68***[0.64, 0.72]
Secondary		0.55*** [0.52, 0.59]			0.63***[0.59, 0.68]
Higher		0.48*** [0.40, 0.57]			0.59***[0.49, 0.70]
**Current working status**					
No		1.00 [1.00, 1.00]			1.00 [1.00, 1.00]
Yes		0.82*** [0.79, 0.86]			0.80***[0.77, 0.84]
**Marital status**					
Single		1.00 [1.00, 1.00]			1.00 [1.00, 1.00]
Married		1.10** [1.02, 1.18]			1.06 [0.99, 1.15]
**Number of antenatal care visits**					
0		1.00 [1.00, 1.00]			1.00 [1.00, 1.00]
1–3		0.79*** [0.73, 0.74]			0.82***[0.77, 0.88]
4 or more		0.68*** [0.64, 0.73]			0.71***[0.66, 0.77]
**Place of delivery**					
Home		1.00 [1.00, 1.00]			1.00 [1.00, 1.00]
Health facility		0.77*** [0.74, 0.82]			0.80***[0.76, 0.84]
Other		0.74** [0.64, 0.73]			0.81 [0.65, 1.02]
**Postnatal care visits**					
No		1.00 [1.00, 1.00]			1.00 [1.00, 1.00]
Yes		1.09*** [1.04, 1.14]			1.10***[1.05, 1.15]
**Mass media exposure**					
No			1.00 [1.00, 1.00]		1.00 [1.00, 1.00]
Yes			0.75***[0.71, 0.78]		0.87***[0.83, 0.92]
**Toilet facility**					
Improved			1.00 [1.00, 1.00]		1.00 [1.00, 1.00]
Unimproved			1.44***[1.37, 1.52]		1.20***[1.14, 1.27]
**Drinking water source**					
Improved			1.00 [1.00, 1.00]		1.00[1.00, 1.00]
Unimproved			0.98 [0.94, 1.03]		0.93**[0.89, 0.98]
**Household size**					
Small			1.00 [1.00, 1.00]		1.00 [1.00, 1.00]
Medium			1.11***[1.06, 1.17]		1.01 [0.96, 1.06]
Large			1.35***[1.26, 1.44]		1.03 [0.96, 1.11]
**Cooking fuel**					
Unclean			1.00 [1.00, 1.00]		1.00 [1.00, 1.00]
Clean			0.68***[0.61, 0.75]		0.77***[0.69, 0.86]
**Wealth index**					
Poorest			1.00 [1.00, 1.00]		1.00 [1.00, 1.00]
Poorer			0.89***[0.83,0.94]		0.92*[0.87, 0.98]
Middle			0.94 [0.88, 1.00]		1.00 [0.94, 1.07]
Richer			0.99 [0.93, 1.07]		1.10**[1.03, 1.19]
Richest			1.02 [0.94, 1.11]		1.27***[1.16, 1.40]
**Place of residence**					
Urban				1.00 [1.00, 1.00]	1.00 [1.00, 1.00]
Rural				1.43***[1.36, 1.51]	1.07*[1.01, 1.14]
**Subregions**					
West				1.00 [1.00, 1.00]	1.00 [1.00, 1.00]
East				0.56***[0.54, 0.59]	0.68***[0.64, 0.72]
Central				0.92**[0.88, 0.98]	0.98 [0.92, 1.04]
Southern				0.50***[0.42, 0.59]	0.80*[0.67, 0.96]
**Random effect result**					
PSU variance (95% CI)	0.020 [0.011, 0.035]	0.016 [0.008, 0.032]	0.018 [0.010, 0.033]	0.021 [0.012, 0.036]	0.017 [0.009, 0.033]
ICC	0.006	0.005	0.005	0.006	0.005
LR Test	16.11 (<0.001)	11.24 (<0.001)	13.29 (<0.001)	17.14 (<0.001)	12.41 (<0.001)
Wald chi-square	Reference	3086.69***	723.34***	705.46***	3360.41***
Model fitness					
Log-likelihood	-31566.216	-29934.673	-31186.784	-31194.6	-29762.046
AIC	63136.43	59915.35	62397.57	62401.2	59598.09
N	110497	110497	110497	110497	110497
Number of clusters	1608	1608	1608	1608	1608

aOR adjusted odds ratios; CI Confidence Interval;

* *p* < 0.05,

** *p* < 0.01,

*** *p* < 0.001

1 = Reference category PSU = Primary Sampling Unit; ICC = Intra-Class Correlation; LR Test = Likelihood ratio Test; AIC = Akaike’s Information Criterion

## Discussion

The study examined the association between birth weight and nutritional status as well as factors associated with undernutrition among children under five years using data from DHS conducted in 32 countries in SSA. The prevalence of low birth weight was 5.4%. The prevalence of low birth weight recorded in this study is lower compared to what was found in other previous studies in SSA (9.5%) [31], Iran (9%) [32], Ghana (9.7%) [33] and Nepal (9.4%) [34]. This finding could be attributed to the variations in study population and design. Moreover, country variations regarding the prevalence of low birth weight were observed. While South Africa had the highest (13.1%) prevalence, Chad recorded the lowest (0.9%). A possible reason for this finding could be the variations in nutritional, antenatal care attendance, and socio-cultural practices among the countries. For example, in South Africa, a lower proportion of women attend 5 or more antenatal care visits [35] and this could increase their chances of giving birth to low birth weight babies.

The study also found that the prevalence of wasting, underweight, and stunting to be 8.1%, 17.0%, and 31.3%, respectively. The prevalence of undernutrition (i.e., wasting, stunting, and underweight) recorded in this study is similar to what was reported in a previous study [12], which found that the prevalence of wasting, stunting, and underweight were 7.1%, 33.2%, and 16.3% respectively. However, compared to another study in Bangladesh [20], this study has a relatively lower prevalence of undernutrition. The highest prevalence of wasting (21.5%) and underweight (37.1%) were found in Niger while the highest prevalence of stunting (51.7%) was found in Burundi. A similar observation was made in another previous study in SSA [12]. This finding could be that the countries (Niger and Burundi) are faced with great geographical disadvantages in accessing arable land for agricultural purposes [36]. It could also be that the countries encounter issues of adverse climatic conditions (e.g., desertification, hot temperatures) that make it impossible for the successful farming related activities and limited food supply [37]. The finding of the study indicates that undernutrition among children under five years in countries in SSA persists with contextual variations. Therefore, policymakers should direct more efforts in the development and implementation of nutrition-sensitive programs (e.g., complimentary feeding, dietary supplementation) that will help reduce or at best eliminate the problem in countries in SSA [38].

Similar to other previous studies [20–22, 24, 25], this study found that children with low birth weight were more likely to be stunted. One of the major factors that account for low birth weight among children in developing countries is intra-uterine growth retardation (IUGR) [39]. Usually, babies who suffer from IUGR are medically born to be malnourished [20]. Research has shown that most of the cases of IUGR in developing countries emanate from mother’s malnutrition status, low maternal weight, and stature during conception [40]. Iron deficiency has been found to have a linkage with IUGR, hence with low birth weight [41]. Another possible reason for the finding could be that mothers do not provide children with the appropriate food supplementation as children transition from the exclusive breasfeeding stage to the weaning stage where complementary foods should be provided [22]. This finding indicates that parents still need to be educated about the need to provide children with complementary foods as the children are weaned from the breast. This will help reduce the rate of stunting among children under five years in SSA.

Consistent withfindings of previous studies [20, 21, 38, 42], this study found that children with low birth weight were more likely to be underweight. Children with low birth weight could be prone to contracting diseases and infections such as diarrhea, anemia, and respiratory infections, thereby increasing their likelihood of becoming underweight [20]. Also, the low utilization of maternal health services such as antenatal care and postnatal care could contributed to the observed association found in this study [38]. This finding suggests that there is a strong association between low birth weight and underweight. Therefore, policy makers should intensify efforts to reduce the rate of low birth rate among children under five years through education as this will help address underweight among under five children in SSA.

Similar to other previous studies [20, 42], this study found that children with low birth weight were more likely to be wasted. A plausible reason for this finding could be that there is a change in nutritional pattern in recent years as a result of the economic instabilities in countries in SSA, increasing the rate of nutritional problems [42]. The present association could be that the mothers are not empowered, therefore, their ability to cater for their nutritional needs and that of their children is curtailed, making them give birth to children with low birth weight which is directedly linked to wasting [43]. This finding indicates that mothers should be empowered before, during, and after birth in order for them to be able to provide the appropriate nutrition for their children as this will help reduce wasting among children under five years in SSA.

### Strengths and limitations

This was a comprehensive study that showed the association between low birth weight and undernutrition indices among children under-five across 32 countries in SSA. The current study provides useful epidemiological insights and add to existing literature on child nutrition. Given the use of nationally representative datasets and the use of a robust statistical method that considered individual, household and contextual level factors, findings are important and generalizable. However, due to the cross-sectional nature of the design, causal link on the associations noted cannot be established. The time when the surveys were conducted varied by up to nine years across studied countries and needs to be considered as it may affect the comparisons due to the time effect.

## Conclusions

Our study has demonstrated that low birth weight is a key determinant for undernutrition among children under-five in SSA. Current findings point to the need for policy makers to give special attention to improving the nutritional status of children under five in SSA by implementing measures (e.g., regular nutritional counselling) and other nutrition-sensitive interventions (e.g., complementary feeding, nutritional supplementation, dietary diversity initiatives) aimed at enhancing the weight of children. Empowering women could also play a significant role in fighting undernutrition among children under five years in SSA. Future research could target intervention programmes to address low birth weight and undernutrition among children under five in SSA countries using longitudinal designs.
